# Review of Bone Conduction Hearing Devices

**DOI:** 10.3390/audiolres11020019

**Published:** 2021-05-18

**Authors:** Susan E. Ellsperman, Emily M. Nairn, Emily Z. Stucken

**Affiliations:** Department of Otolaryngology—Head and Neck Surgery, University of Michigan, Ann Arbor, MI 48109, USA; emnairn@med.umich.edu (E.M.N.); estucken@med.umich.edu (E.Z.S.)

**Keywords:** bone conduction, bone-anchored hearing aid, osseointegrated implant, transcutaneous bone conduction, percutaneous bone conduction

## Abstract

Bone conduction is an efficient pathway of sound transmission which can be harnessed to provide hearing amplification. Bone conduction hearing devices may be indicated when ear canal pathology precludes the use of a conventional hearing aid, as well as in cases of single-sided deafness. Several different technologies exist which transmit sound via bone conduction. Here, we will review the physiology of bone conduction, the indications for bone conduction amplification, and the specifics of currently available devices.

## 1. Introduction

The concept of bone conduction hearing, the phenomenon through which a vibrating object can transmit sound, was first described in writing in the 1500s and credited to Girolamo Cardano [1]. Rudimentary devices such as a rod or spear were initially utilized as assistive devices for those with hearing loss by providing a route for vibrations to reach the listener. As technology advanced and the carbon microphone was developed in the early 1900s, bone conduction devices designed to convert sounds into mechanical signals that vibrate the mastoid bone were created. Early devices were held in place with a headband or eyeglasses and proved to be beneficial despite the cumbersome design and inefficient sound transmission. These early investigations paved the way for the development of modern bone-anchored hearing aids surgically implanted into the temporal bone. In 1977, Anders Tjellström and his colleagues in Sweden were the first to implant a percutaneous titanium device utilizing an osseointegrated screw [2]. The concept of osseointegration, direct contact between living osteocytes and the titanium implant, was developed by Brånemark and initially utilized for dental implants [3]. The first bone-anchored hearing device became widely commercially available in the 1980s, and since that time, patients with conductive hearing loss (CHL), mixed hearing loss (MHL), and unilateral hearing loss or single-sided deafness (SSD) have benefitted from these devices [4]. This review aims to provide an overview of bone conduction hearing physiology and the currently available bone conduction hearing devices including the indications, fitting range, benefits, and drawbacks of each.

## 2. Bone Conduction Physiology

Multiple physiologic mechanisms contribute to bone conduction hearing. Put simply, sound energy is transmitted from vibrations in the skull to the cochlea, which ultimately results in wave propagation along the basilar membrane and stimulation of the cochlear nerve—the same endpoint as air conduction hearing [5]. There is ongoing investigation to fully describe the mechanisms by which bone conduction hearing occurs and the relative contributions of each pathway. Five major pathways were well summarized by Stenfelt and Goode in 2005 [6]. In their review of previously published data and their own findings, they describe (1) sound radiation to the external ear canal, (2) middle ear ossicle inertia, (3) inertia of cochlear fluids, (4) compression of the cochlear walls (or inner ear compression), and (5) pressure transmission from cerebrospinal fluid (CSF) as the principal contributors to bone conduction. Inertia of cochlear fluids is felt to be the most important contributor [6].

Bone conduction hearing aids take advantage of the above mechanisms by converting sound energy into skull vibrations. Since the initial work by Tjellström [2] and his colleagues, there have been numerous commercial devices introduced, including surgically implanted and extrinsically applied devices. These devices are intended to assist with hearing rehabilitation for patients with conductive or mixed hearing loss who are unable to utilize conventional air conduction hearing aids or for patients with single-sided deafness. The ability to use conventional, transcanal devices may be limited by recurrent infections such as chronic otitis externa, prior surgical intervention and altered anatomy, microtia or anotia, canal atresia or stenosis, or other anatomic constraints. In the single-sided deafness population, bone conduction devices route signals transcranially to the contralateral, normal hearing cochlea.

When choosing a bone conduction device, many factors must be considered. Each patient has unique needs which are related to their degree and type of hearing impairment, anatomy, vocational or educational needs, and personal preferences. Finding this information in a consolidated location can be challenging for patients and providers. The goal of this review is to provide an overview of the current device landscape including the hearing losses best treated by each device, surgical and nonsurgical advantages and disadvantages for each class of devices, magnetic resonance imaging (MRI) compatibility, processor characteristics, wireless connectivity, and available accessories. The following description of devices includes products currently available and utilized in the United States. While meant to be inclusive of all manufacturers and products, devices in the ever-evolving landscape may have been inadvertently excluded or developed following the preparation of this review.

## 3. Currently Available Devices

### 3.1. Surgically Implanted Devices

Surgically implanted bone conduction devices convert acoustic sound waves into mechanical vibration, which is conducted to the inner ear via direct contact with the skull. These can be classified broadly into percutaneous and transcutaneous devices based on the presence or absence of a skin-penetrating abutment. The transcutaneous devices can be further classified into active and passive implants. Passive transcutaneous devices have an implanted portion of the device in direct connection with the skull and a separate, external portion held in place magnetically which drives vibration through the skin to the implanted device. In a passive system, vibration occurs at the level of the external processor, and vibrations are transmitted transcutaneously to the implanted device. Active transcutaneous devices contain an external microphone and processor which send electronic signals to an implanted, vibrating device in direct contact with the skull. With an active system, the external processor is static and transmits electronic signals. Vibration occurs at the level of the implanted device only. Currently available devices including indications for the selection of each, benefits, and drawbacks will be discussed.

#### 3.1.1. Percutaneous Devices

Direct contact with the skull affords a meaningful advantage for percutaneous devices over passive transcutaneous devices. Passive transcutaneous devices rely on vibratory signal delivery through the skin and are subject to signal attenuation up to 20 dB, especially at high frequencies [7]. The direct connection of the percutaneous devices allows for efficient signal transmission at all frequencies without skin and soft tissue impedance. Surgical insertion of percutaneous devices is performed under local or general anesthesia through a variety of skin incisions [8]. Single-stage procedures are now standardly utilized except in situations with concern for poor wound healing or poor bone mineralization in which a two-stage operation may be considered. Traditionally, the sound processor is activated and loaded onto the abutment three months post-operatively, but the recent literature has examined the role for earlier activation at one to two weeks, or even one day post-operatively without sacrificing implant stability [9,10,11].

The most significant disadvantage of percutaneous implants is the potential for adverse skin reactions, device extrusion, and the need for revision surgery. The reported complication rate varies widely and appears to be influenced by the surgical technique, surgeon experience, patient age, and patient factors predisposing to infection or poor wound healing. Surgery for the placement of a percutaneous abutment was often performed with skin grafting in the past; however, skin grafting is no longer performed regularly which has resulted in overall improved cosmesis with fewer graft complications. Adverse skin reactions continue to be the most common complication of percutaneous devices, and can be categorized using the Holgers classification, a scale from zero to four described in Table 1 [12]. A 2016 systematic review published by Mohamad et al. included 30 published studies and cites a skin complication rate ranging from 9.4 to 84% [13]. A 2013 meta-analysis by Kiringoda and Lustig included 2310 implants and cited a rate of grade 2 or higher skin complications ranging from 2.4 to 38.1% [14]. The rate of revision surgery ranged from 1.7 to 34.5% in adult or mixed populations and 0 to 44.4% in pediatric populations [14].

Currently available percutaneous bone conduction devices include the Oticon Ponto System (Oticon Medical AB, Askim, Sweden) [15] and the Cochlear^TM^ Baha^®^ Connect System (Cochlear Bone-Anchored Solutions AB, Mölnlycke, Sweden) [16,17]. In general, these devices consist of an osseointegrated implant (screw), skin penetrating abutment, and an external sound processor. The implant and abutment may be coupled and implanted together. The devices are recommended for patients with MHL, CHL, or SSD. The degree of accepted sensorineural hearing loss varies depending on the power of the processor. In patients with a purely conductive hearing loss, those with an air–bone gap of at least 30 dB are more likely to benefit from a bone-anchored device compared to a traditional air conduction aid [18]. Patients with SSD should have a pure tone average (PTA) of better than or equal to 20 dB hearing level (HL) in the contralateral, normal hearing ear.

**Figure 1:**
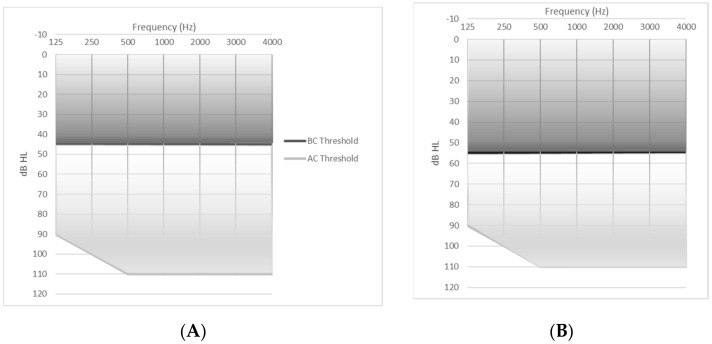

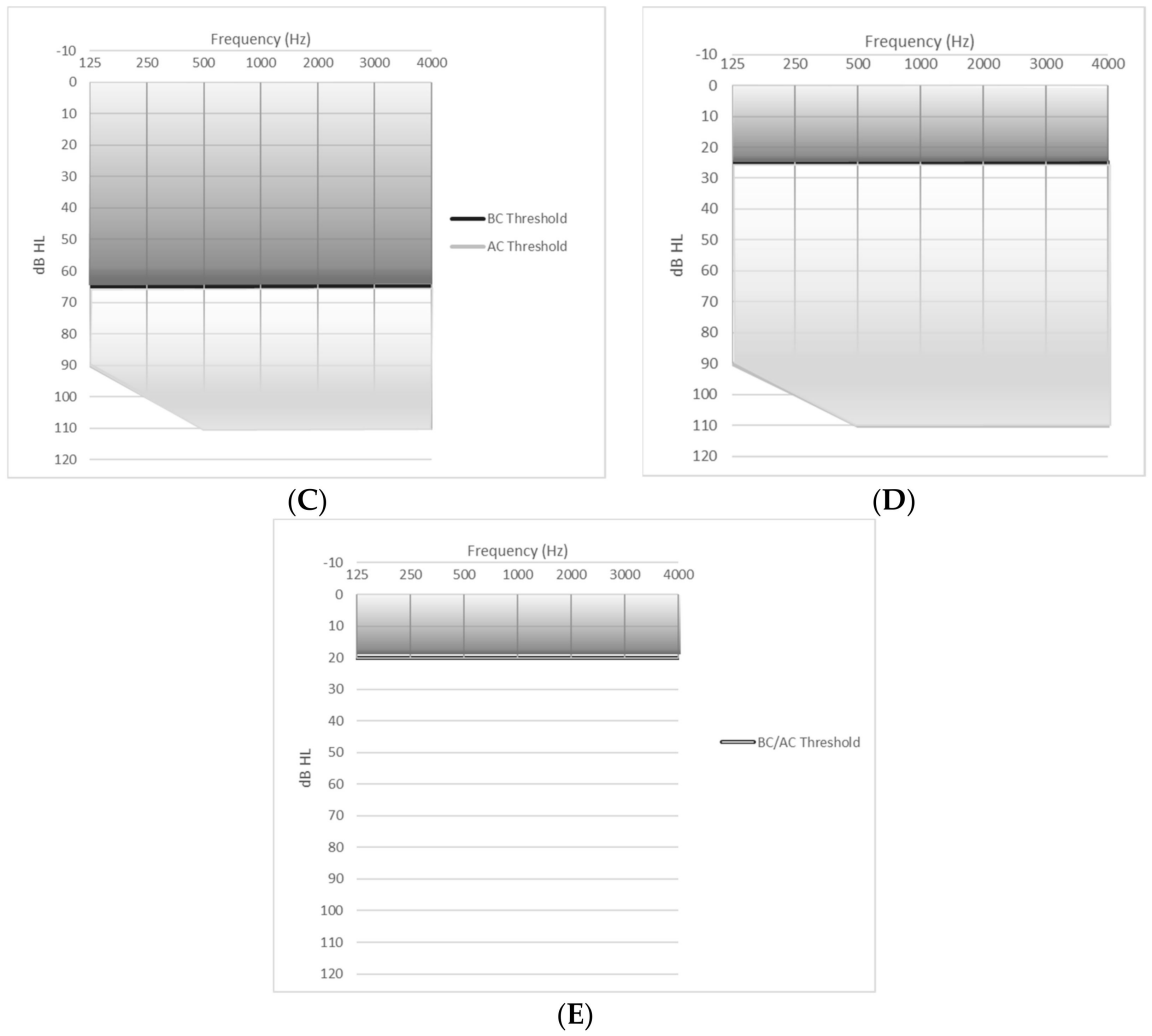
This figure depicts the fitting ranges for the described devices. The dark grey shaded area represents the range of recommended bone conduction thresholds in patients being considered for bone conduction hearing devices. The light grey shaded area demonstrates possible air conduction thresholds. (**A**) represents a 45 dB BC PTA, the recommended bone conduction hearing threshold for the Ponto 3 [15], Ponto 4 [15], Baha^®^ 5 [17], Alpha 2 MPO ePlus^TM^ [42], and SAMBA 2 [30] processors. (**B**) represents a 55 dB BC PTA, the recommended bone conduction hearing threshold for the Ponto 3 Power [15], Baha^®^ 5 Power [17], Baha^®^ 6 Max [16], and Osia^®^ 2 [25] processors. (**C**) represents a 65 dB BC PTA, the recommended bone conduction hearing threshold for the Ponto 3 SueprPower [15] and the Baha^®^ 5 SuperPower [17]. (**D**) represents a 25 dB BC PTA, the recommended bone conduction hearing threshold for the ADHEAR processor [31]. (**E**) represents a 20 dB BC PTA. For patients with SSD, the contralateral ear should have normal hearing—a BC and AC PTA of 20 dB or better. These figures were created from publicly available device information and reproduced with permission from Cochlear^TM^, MED-EL, Medtronic, and Oticon representatives.

The Oticon Ponto became commercially available in 2009. The currently utilized implant is a 4.5-mm-wide, 6 mm long, titanium screw with an abutment [19]. Currently available processors include the Ponto 3 and Ponto 4 series devices. The Ponto 3 has three versions: Ponto 3, Ponto 3 Power, and Ponto 3 SuperPower. These processors are intended for patients with bone conduction hearing thresholds up to 45 dB HL, 55 dB HL, and 65 dB HL, respectively (Table 2; Figure 1). The Ponto 4 is a smaller device and suitable for bone conduction hearing thresholds up to 45 dB HL (Table 2; Figure 1) [15].

The Cochlear^TM^ Baha^®^ Connect System utilizes the BI300, a titanium osseointegrated implant which is available in 3- or 4-mm lengths. The percutaneous abutment, the BA400, is hydroxyapatite-coated and is available in 6-, 8-, 10-, 12-, and 14-mm lengths to accommodate a range of soft tissue thickness [32]. The currently available series includes the Baha^®^ 5, Baha^®^ 5 Power, and the Baha^®^ 5 SuperPower sound processors. These devices are intended for patients with bone conduction hearing thresholds up to 45 dB HL, 55 dB HL, and 65 dB HL, respectively (Table 2; Figure 1) [17]. To achieve a higher output, the Baha^®^5 SuperPower has a behind-the-ear component to allow for the physical separation of the actuator from the microphone [17]. The Baha^®^ 6 Max was recently FDA-approved and suitable for bone conduction hearing thresholds up to 55dB HL and is anticipated to be commercially available soon (Table 2; Figure 1) [16]. 

The SuperPower processors for the Ponto and Baha^®^ systems each provide powerful processors intended for patients with bone conduction hearing thresholds up to 65 dB HL. The systems have some differences that impact the fitting and use of the processors. The Ponto 3 SuperPower is one piece and less bulky than the Baha^®^ SuperPower processor [15,17]. Feedback may be harder to control due to the inability to separate the actuator from the microphone. In contrast, the Baha^®^ 5 SuperPower system allows for the separation of the actuator from the microphone and can be worn in several configurations for even greater separation if feedback or physical placement becomes an issue [15]. This system is larger, with two pieces, and bulkier than the Ponto 3 SuperPower device. Placement of the larger device may be challenging in patients who were initially implanted in anticipation of a standard processor but have converted to a SuperPower processor to address the worsening of sensorineural hearing. The implant placement in these patients may not be ideal to accommodate the bulkier SuperPower processor. The Baha^®^ 5 SuperPower processor uses rechargeable batteries similar to a cochlear implant (Table 3) [17]. Available accessories and streaming capabilities are listed in Table 4.

#### 3.1.2. Passive Transcutaneous Devices

Transcutaneous systems were designed to avoid the cosmetic concerns and skin complications associated with percutaneous devices while still delivering adequate sound transmission. In the transcutaneous systems, a titanium implant is placed directly in the skull in the same manner as the percutaneous devices. A magnet is attached to this implant, and the skin is closed over the top of the implant, avoiding a percutaneous component. Once the incision has healed and osseointegration has occurred, the external device is then activated. The external device is retained via attraction to the internal magnet and vibrates in response to sound inputs. The vibratory force then passes through the intervening skin and soft tissue to reach the internal magnet and implant which allow the transmission of the vibration to the skull.

While skin complications are less common than those seen with percutaneous devices, the magnetic force required to hold the external device in place and effectively transmit sound in transcutaneous systems can lead to pain and irritation of the intervening skin and soft tissue. When this occurs, the magnet strength can be reduced to decrease the amount of pressure applied to the skin, and users may be instructed to reduce daily wearing time or avoid using their device altogether until symptoms improve. If the amount of pressure applied is greater than the patient’s capillary pressure, the skin may have inadequate blood supply and necrosis can occur [33]. A systematic review by Cooper et al. reported a 13.1% rate of minor soft tissue complications which resolved spontaneously or with use of a weaker magnet [34]. A grading system comparable to the Holgers scale for percutaneous implants has not been established; thus, reporting and comparing skin complications is challenging [12]. The rate of major complications, defined as complications requiring active management, such as post-operative seroma, hematoma, wound infections, skin ulcerations, and dehiscence, was 5.2% in the same systematic review [34].

The Baha^®^ Attract System (Cochlear Bone-Anchored Solutions AB, Mölnlycke, Sweden) [35] and Alpha 2 MPO (formerly SOPHONO^TM^) system (Medtronic, Dublin, Ireland) [23] are the available passive transcutaneous devices. Both devices are intended for the treatment of CHL, MHL, or SSD with normal contralateral hearing. While auditory outcomes have shown significant improvement compared to unaided conditions, signal attenuation occurs due to signal loss during transmission through the skin and soft tissues [36]. This attenuation is most apparent at high frequencies and may be as high as 25 dB at 6000 to 8000 Hz higher frequencies when compared to percutaneous devices [37,38].

The Baha^®^ Attract uses the same BI300 implant as the percutaneous Baha^®^ Connect. During insertion of the device, bone polishing is performed if needed to accommodate the attachment of the BIM400 implant magnet to the BI300 without the magnet making direct contact to the bone [35]. The thickness of the skin flap over the magnet must be 6mm or less, which at times may require soft tissue reduction [35]. The Baha^®^ Attract utilizes the same external processors as the Baha^®^ Connect intended for use with the same bone conduction hearing thresholds previously listed (Table 2; Figure 1) [16,17]. The external processors are attached to a magnet rather than directly articulating to the percutaneous post. Once adequate healing and osseointegration have taken place, the external sound processor and magnet are applied and activated. Users are instructed to begin by wearing the device a few hours a day and slowly increase usage over time to avoid skin irritation and limit discomfort. The application of a SoftWear^TM^ pad as a barrier between the skin and device is recommended by the manufacturer [39]. Six magnets of increasing strength are available to accommodate for variable soft tissue thickness, overlying hair, and patient comfort [39]. Since the Baha^®^ Attract and Connect devices use the universal BI300 implant, it is possible to convert from a Baha^®^ Connect to a Baha^®^ Attract device, though the skin at the previous abutment site must be healed and healthy prior to conversion [40,41]. The Baha^®^ Attract is MRI compatible at 1.5 Tesla with the internal magnet in place. A sizeable area of artifact will be present on the MRI, which is significantly larger than the degree of artifact with percutaneous devices. The magnet may be surgically removed if a higher strength MRI is required or if the resultant artifact obscures critical image sequences (Table 3) [24]. Available accessories and streaming capabilities are listed in Table 4.

The Alpha 2 MPO implant system consists of two internal magnets hermitically sealed in a titanium case. This device is designed to sit within shallow bone beds which are drilled based on manufacturer recommendations. The Alpha 2 MPO device is then attached to the skull with five screws [34,37]. The Alpha 2 MPO ePlus^TM^ sound processor is then applied and drives vibrations through the skin and soft tissue using transcutaneous energy transfer or TET^TM^. The device is approved for patients with up to a 45 dB hearing loss with ideal candidacy up to 35 dB HL (Table 2; Figure 1) [42]. The Alpha 2 MPO system is MRI compatible up to 3 Tesla (Table 3) [28]. Available accessories and streaming capabilities are listed in Table 4.

#### 3.1.3. Active Transcutaneous Devices

Active transcutaneous bone conduction devices were designed to maximize the benefits of percutaneous and passive transcutaneous devices while avoiding skin complications and soft tissue signal attenuation. Active devices have an external processor and implanted transducer which are connected by magnetic coils. Signals are transmitted electrically from the external to internal device using technology akin to that of cochlear implants. As the internal device is responsible for generating mechanical forces against the skull, skin attenuation does not occur, and magnet strength can be significantly reduced.

Available devices include the Bonebridge^TM^ (MED-EL, Innsbruck, Austria) [29], and the recently introduced Osia^®^ 2 System (Cochlear Bone-Anchored Solutions AB, Mölnlycke, Sweden) [25]. The Bonebridge^TM^ was first introduced in 2012 with the second version, the BCI602, released in 2019. The device is indicated for patients with CHL, MHL with BC PTA thresholds better than or equal to 45 dB HL, or SSD (Table 2; Figure 1). The implanted device consists of a magnet, receiving coil, demodulator which processes sounds, and an electromagnetic floating mass transducer (FMT) which generates mechanical vibrations [29]. The FMT is attached to the skull via cortical fixation screws that do not require osseointegration [43]. The BCI602 requires a bony recess drilled into the skull, though the BCI602 is smaller in size than the original implant making placement more straightforward. Optimal placement is in the pre-sigmoid mastoid bone. In patients that have had a prior mastoidectomy, alternative placement in a retrosigmoid position or above the temporal line may be required. The device has a flexible bridge between the receiver coil and the FMT to allow for greater flexibility in placement when needed. Lifts are available to limit the amount of required bone excavation and separate the device from underlying dura or sinuses [44,45]. Preoperative CT imaging is recommended [45]. The external processor is the SAMBA 2 processor which is held in place magnetically with six magnet strengths available [30]. With the external processor removed, this device is MRI compatible up to 1.5 Tesla (Table 3) [29]. 

The Osia^®^ System was introduced in the United States in 2019 and indicated for patients with CHL, MHL with BC PTA thresholds of 55 dB HL or better, and SSD (Table 2; Figure 1) [25]. The system uses the same BI300 osseointegrated implant as other Cochlear^TM^ devices with the OSI200 implant fixated to the osseointegrated BI300 screw [26]. Bone polishing may be required to ensure the transducer is in contact with the implant only and not surrounding bone, but drilling a bony well is not required [46]. This device uses a piezoelectric transducer which undergoes mechanical deformation when an electric voltage is applied [47]. This motion drives vibration through the BI300 implant to the skull, allowing for bone conduction hearing. The current device is not MRI compatible; the implanted magnet must be surgically removed before an MRI can be safely performed (Table 3) [27]. Available accessories and streaming capabilities are listed in Table 4.

### 3.2. Extrinsic Devices

Non-surgical bone conduction hearing devices are also available. These are attached to the patient via a headband, softband, adhesive, eyeglasses, or another mechanism. The external device is in contact with the skin, vibrates in response to sound, and transmits vibratory signals through the intact skin and soft tissue to the skull, leading to bone conduction hearing. These devices are subject to signal attenuation, especially at high frequencies, as the signal travels through soft tissue [7]. Depending on the attachment mechanism, the force required to hold the device in place and effectively transmit sound may limit wear time [48]. The same bone-anchored hearing processors used in the transcutaneous passive devices can be attached to a test band. Pre-implantation testing is recommended for patients considering bone-anchored hearing aid placement to help patients understand the benefits of such devices, sound quality, and the utility of choosing a bone conduction device.

Similar to passive transcutaneous devices, signal attenuation, especially at high frequencies, is expected [7]. Percutaneous or active transcutaneous devices would be expected to perform better, but the trial period allows patients to make a more informed decision about proceeding with surgery and the hearing quality they can anticipate post-operatively. Bone-anchored hearing aid placement is currently FDA-approved for children five years of age or older [49]. Children too young for implantation or adult patients for whom surgery is contraindicated may use a headband device as for amplification beyond the trial environment.

Previously introduced processors including Cochlear^TM^ Baha^®^ 5 series, Ponto 3 and 4 series, and Alpha MPO ePlus^TM^ devices can all be worn externally on a soft band, headband, or other attachment mechanism. Two devices may be worn when bilateral amplification is indicated.

In addition to these devices, an adhesive option, the ADHEAR (MED-EL, Innsbruck, Austria) is also available [31]. This device is anchored with an adhesive applied to the skin over the mastoid bone which is designed to be worn for three to seven days. The audio processor connects to the adhesive and vibrates in response to sound, driving vibratory signal transmission through the skin and soft tissue to the underlying bone [31]. Since it is attached by an adhesive, pressure-induced discomfort is not a limitation to wear [48]. The ADHEAR is indicated for patients with unilateral or bilateral conductive hearing loss with a bone conduction HL better than or equal to 25 dB and for patients with single-sided deafness (Table 2; Figure 1) [31]. Available accessories and streaming capabilities are listed in Table 4.

The SoundBite (Sonitus Technologies, San Mateo, CA, USA) is a dental appliance designed to transmit vibratory signals to the skull via the teeth [50,51]. The device is not currently available, but a brief discussion is included here for reference. The device was designed for patients with single-sided deafness or conductive hearing loss with a bone conduction PTA better than or equal to 25 dB HL [20]. The SoundBite^TM^ consists of an in-the-mouth (ITM) piezoelectric transducer placed on the buccal surface of the maxillary molars and a device worn on the poorer hearing ear which consists of a behind-the-ear (BTE) transducer and a microphone in the ear canal [51]. This has been found to be safe and uses forces far below those typically felt by the teeth during normal functions [50]. Production of the device stopped in 2015, but Sonitus Technologies was recently awarded a contract with the United States Department of Defense with the plan to rebrand the device as the Molar Mic^TM^ for military personnel [52].

**Table 3:** Sound Processor Characteristics.

	Device	Processor	Size	Weight	Battery Type	Average Battery Life	IP Rating [53]
Percutaneous	Ponto [15,21]	Ponto 3	3.4 × 2.1 × 1.4 cm	14 g (without battery)	13	70–130 h	IP 57
		Ponto 3 Power		17 g (without battery)	675	70–150 h	IP 57
		Ponto 3 Superpower		17 g (without battery)	675 HP	35–80 h	IP 57
		Ponto 4	2.6 × 1.9 × 1.1 cm	13.2 g (without battery)	312	48–70 h	IP 57
	Baha^®^ Connect [16,17,54,55]	Baha^®^ 5	2.6 × 1.9 × 1.2 cm	9.8 g (without battery)	312	36–100 h	IP 63
		Baha^®^ 5 Power	3.6 × 2.2 × 1.3 cm	17 g (without battery)	675	80–220 h	IP 63
		Baha^®^ 5 SuperPower	3.9 × 4.8 × 0.9 cm	14.4 g (actuator); 9.8 −12.7 g (processing unit + battery)	Rechargeable lithium	≤16 h (mini) ≤32 h (standard)	IP 63
		Baha^®^ 6 Max	2.6 × 1.9 × 1.2 cm	11.5 g (without battery)	312	44–132 h	IP 68
Transcutaneous Passive	Alpha 2 MPO [23]	Alpha 2 MPO ePlus^TM^	4.1 cm × 1.63 cm		13 or rechargeable	320 h or 32 h (rechargeable)	IP 22
	Baha^®^ Attract [16,17,24]	Same as Baha^®^ Connect	Same as above				
Transcutaneous Active	Osia^®^ [22,25,26,27]	Osia^®^ 2	3.6 × 3.2 x 1.04	7.8 g (with magnet; without battery)	675 HP		IP 52; IP 68 (with cover)
	Bonebridge^TM^ [28,29,30]	SAMBA 2	3.0 × 3.5 × 1.0 cm	7.5 g (with magnet; without battery) *	675	133–210 h	IP 54; IP 68 (with cover)
Adhesive	ADHEAR [31]	ADHEAR	0.6 × 3.0 cm (adhesive) 1.5 × 3.5 cm (processor)	13.5 g (without battery)	13	Up to 300 h	

Device characteristics and compatibility for each processor are listed including external processor size, weight, battery type, battery life, and IP (ingress protection) rating. IP Rating = “ingress protection” rating, indicates the amount of resistance to solids and liquids. The first number indicates the amount of resistance to solids (with 0 being not protected, and 6 being dust-tight), and the second digit indicates the amount of resistance to liquids (with 0 being not protected, and 8 being protected from liquids up to 1m of submersion) [55]. Device information is included with permission from Cochlear^TM^, MED-EL, Medtronic, and Oticon representatives. Note that battery life is variable depending on the programs and features utilized and streaming time. (HP = high power battery type). * D. Franz, email communication, April 2021.

**Table 4:** Sound Processor Connectivity and Accessories.

	Device	Processor	Wireless Accessories	Streaming Method	Direct iPhone Streaming	Direct Android Streaming
Percutaneous	Ponto [15,53,56]	Ponto 3	Ponto 3 Connect Line App Oticon Medical Streamer on neck loop Remote Mic TV Adapter 2.0 FM system compatible Phone adapter 2.0 BTD 500 Ponto 4 Oticon ON app Remote Control 3.0 Connect Clip (can be used as a remote mic) TV Adapter 3.0 Edumic Phone adapter 2.0 Bluetooth dongle BTD 800	NFMI on neck loop; 2.4 GHz to devices		
		Ponto 3 Power				
		Ponto 3 Superpower				
		Ponto 4		2.4 GHz	X	
	Baha^®^ Connect [16,17,21,54,55,57]	Baha^®^ 5, Baha^®^ 5 Power, and Baha^®^ 5 SuperPower	Baha 5 and 6 Baha^®^ Smart App Remote Control 2 Mini Microphone 2+ Phone Clip TV Streamer	2.4 GHz	X	
		Baha^®^ 6 Max		2.4 GHz; Bluetooth LE	X	X
Transcutaneous Passive	Alpha 2 MPO [23]	Alpha 2 MPO ePlus^TM^	None Note that DAI can be used for wired streaming and FM systems	DAI		
	Baha^®^ Attract [7,16,17,24]	Baha^®^ 5, Baha^®^ 5 Power, and Baha^®^ 5 SuperPower; Baha^®^ 6 Max	Same as above			
Transcutaneous Active	Osia^®^ [25,26,27,58]	Osia^®^ 2	Osia^®^ Smart App TrueWireless^TM^ Phone Clip Mini mic 2 Remote control 2 TV streamer	2.4 GHz	X	
	BONEBRIDGE^TM^ [9,28,29,30,59]	SAMBA 2	SAMBA2GO SAMBA 2 Remote App Note that DAI can be used for wired streaming and FM systems	NFMI on neck loop; Bluetooth or DAI to devices		
Adhesive	ADHEAR [31,60]	ADHEAR	None Note that DAI can be used for wired streaming and FM systems	DAI		

2.4 GHz = The 2.4 GHz Industrial Scientific Medical (ISM) band is similar to Bluetooth streaming and allows wireless signal to propagate through the air to connect/stream with the hearing processor. NFMI = near field magnetic induction; BT LE = Bluetooth low energy; Bluetooth technology that utilizes the traditional “frequency-hopping” 2.4 GHz band technology, but requires less energy consumption. Best for devices in short range of each other [61]. Device information is included with permission from Cochlear^TM^, MED-EL, Medtronic, and Oticon representatives.

## 4. Conclusions

Since the introduction of bone conduction hearing technology, numerous devices have been developed to optimize signal transmission, limit skin and wound complications, and rehabilitate hearing for patients with conductive and mixed hearing loss and single-sided deafness. The recently introduced active transcutaneous devices, the Osia^®^ and Bonebridge^TM^, take advantage of new electronic signal transmission, optimize bone conduction efficiency, and reduce the incidence of skin complications. The current landscape of devices is described here and includes fitting criteria, patient selection, and benefits and drawbacks of each device. This condensed information is intended to be a resource for patients and providers alike to assist with proper device selection for each situation.

## Figures and Tables

**Table 1:** Holgers classification of skin complications.

Grade	Description	Management
0	No irritation	Remove epithelial debris if present
1	Slight redness	Local treatment
2	Red and slightly moist tissue (no granuloma)	Local treatment
3	Reddish and moist (may have granulation tissue)	Revision surgery indicated
4	Infection	Removal of skin penetrating implant necessary

The Holgers classification is used to classify and describe skin complications following percutaneous device placement [12].

**Table 2:** Sound processor specifications.

	Device	Processor	Fitting Range	Frequency Range (DIN45.605)	Peak OFL * at 90 dB SPL	Peak OFL * at 60 dB SPL	Processing Delay	MRI Compatibility
Percutaneous	Ponto ^†^ [15,20]	Ponto 3	BC PTA ≤ 45 dB	200–9500 Hz	124 dB	107 dB	6 ms	Compatible up to 3 Tesla
		Ponto 3 Power	BC PTA ≤ 55 dB	260–9600 Hz	128 dB	116 dB	6 ms	
		Ponto 3 Superpower	BC PTA ≤ 65 dB	260–9600 Hz	135 dB	125 dB	6 ms	
		Ponto 4	BC PTA ≤ 45 dB	200–9500 Hz	124 dB	108 dB	8 ms	
	Baha^®^ Connect ^‡^ [16,17,21,22]	Baha^®^ 5	BC PTA ≤ 45 dB	250–7000 Hz	117 dB	105 dB	4.5 ms	Compatible up to 3 Tesla
		Baha^®^ 5 Power	BC PTA ≤ 55 dB	250–7000 Hz	123 dB	113 dB	4.5 ms	
		Baha^®^ 5 SuperPower	BC PTA ≤ 65 dB	250–7000 Hz	133 dB	121 dB	4.5 ms	
		Baha^®^ 6 Max	BC PTA ≤ 55 dB	200–9700 Hz	121 dB	108 dB	<6 ms	
Transcutaneous Passive	Alpha 2 MPO ° [23]	Alpha 2 MPO ePlus^TM^	BC PTA ≤ 45 dB (ideal ≤ 35 dB)	125–8000 Hz	120 dB	110 dB		Compatible up to 3 Tesla
	Baha^®^ Attract ^‡^ [16,17,22,24]	Baha^®^ 5	BC PTA ≤ 45 dB	250–6300 Hz	114 dB	104 dB	4.5 ms	Compatible up to 1.5 Tesla
		Baha^®^ 5 Power	BC PTA ≤ 55 dB	250–7000 Hz	125 dB	115 dB	4.5 ms	
		Baha^®^ 5 SuperPower	BC PTA ≤ 65 dB	250–7000 Hz	134 dB	123 dB	4.5 ms	
		Baha^®^ 6 Max	BC PTA ≤ 55 dB	200–9250 Hz	121 dB	108 dB	<6 ms	
Transcutaneous Active	Osia^^®^ ‡^ [25,26,27]	Osia^®^ 2	BC PTA ≤ 55 dB	400–7000 Hz			<6 ms	No–internal magnet must be removed
	BONEBRIDGE^TM €^ [28,29,30]	SAMBA 2	BC PTA ≤ 45 dB	250–8000 Hz	117 dB		8 ms	Compatible up to 1.5 Tesla
Adhesive	ADHEAR ^€^ [31]	ADHEAR	BC PTA ≤ 25 dB	250–8000 Hz	124 dB		10 ms	Yes–remove external device

This table includes device specifics for each of the processors discussed and includes fitting ranges, frequency ranges, peak output, and MRI compatibility. (OFL = output force level relative to 1 µN on a skull simulator; * OFL may be measured at FOG (full on gain) or RTG (reference test gain), and therefore may not be directly comparable between devices). Device information is included with permission from Cochlear^TM^, MED-EL, Medtronic, and Oticon representatives. ^†^ Oticon Medical AB, Askim, Sweden; ^‡^ Cochlear Bone-Anchored Solutions AB, Mölnlycke, Sweden; ° Medtronic, Dublin, Ireland; MED-EL, Innsbruck, Austria; ^€^ MED-EL, Innsbruck, Austria.
